# Discriminating Natural Image Statistics from Neuronal Population Codes

**DOI:** 10.1371/journal.pone.0009704

**Published:** 2010-03-25

**Authors:** Satohiro Tajima, Masato Okada

**Affiliations:** 1 Department of Complexity Science and Engineering, The University of Tokyo, Kashiwa, Chiba, Japan; 2 Nagano Station, The Japan Broadcasting Corporation, Nagano-City, Japan; 3 RIKEN Brain Science Institute, Wako, Saitama, Japan; The University of Western Ontario, Canada

## Abstract

The power law provides an efficient description of amplitude spectra of natural scenes. Psychophysical studies have shown that the forms of the amplitude spectra are clearly related to human visual performance, indicating that the statistical parameters in natural scenes are represented in the nervous system. However, the underlying neuronal computation that accounts for the perception of the natural image statistics has not been thoroughly studied. We propose a theoretical framework for neuronal encoding and decoding of the image statistics, hypothesizing the elicited population activities of spatial-frequency selective neurons observed in the early visual cortex. The model predicts that frequency-tuned neurons have asymmetric tuning curves as functions of the amplitude spectra falloffs. To investigate the ability of this neural population to encode the statistical parameters of the input images, we analyze the Fisher information of the stochastic population code, relating it to the psychophysically measured human ability to discriminate natural image statistics. The nature of discrimination thresholds suggested by the computational model is consistent with experimental data from previous studies. Of particular interest, a reported qualitative disparity between performance in fovea and parafovea can be explained based on the distributional difference over preferred frequencies of neurons in the current model. The threshold shows a peak at a small falloff parameter when the neuronal preferred spatial frequencies are narrowly distributed, whereas the threshold peak vanishes for a neural population with a more broadly distributed frequency preference. These results demonstrate that the distributional property of neuronal stimulus preference can play a crucial role in linking microscopic neurophysiological phenomena and macroscopic human behaviors.

## Introduction

Understanding how the human visual system recognizes complex natural images is a most important but challenging problem in vision science. In the field of image engineering, a promising first step toward solving this problem is analyzing statistical properties of natural images and making reduced models of them based on their statistical redundancies [1]. For example, the distribution of amplitude across spatial frequency in natural scene imagery is not like white noise but rather follows a well-known ‘power law’ [2]–[7]; that is, the amplitude at spatial frequency 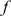 falls by approximately a factor of 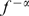 with a particular constant 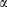 (we call this constant the ‘falloff parameter’). The value of the falloff parameter 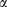 varies among individual images, but typically falls within a range of 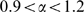 for natural scenes [2]. The formulation based on the ‘power law’ gives reduced descriptions of natural image amplitude spectra, using (for the simplest case) two parameters 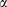 and the image contrast. Relatively recent studies suggest that the shape of the amplitude spectra falloff can characterize not only a whole class of natural image but also its subclasses, which can be determined by image properties such as texture [8] or blurriness [9]. Those careful observations with the modeling and the analysis of natural images suggest that determining the exact 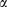 values is not a trivial issue; we can find functional meanings in the values of 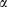 in natural image recognition. In parallel with the modeling work mentioned above, many psychophysical studies have been conducted to ask whether and how natural image statistics are related to human visual performance. Experiments using grayscale natural images or artificial noise have shown that humans can discriminate between images with slightly different 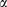 values, and the ability to discriminate varies depending on the values of 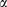 itself [4], [8], [10]–[15]. Roughly, the human sensitivity to the change in the slope of the spectrum falloff is the highest for the images with 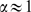, which is typically observed in natural scenes [8], [10]. It can be speculated that that human performance is determined by the resolution of encoding within the visual nervous system. However, a detailed model of the neural processing underlying the discrimination of the image statistics has been poorly studied so far. This study is motivated by the desire to fill this gap between the stimulus features and resulting human performance.

We propose a computational model of neuronal encoding and decoding of natural image statistics. We presume that the spatial-frequency selective neurons, which are observed in the early visual cortex, are the main neural substrate for representing the image statistics. Unlike laboratory stimuli such as sinusoidal grating or the Gabor patch, natural images generally contain signals broadly distributed over different spatial frequencies. Such a broad-band stimulus would activate a relatively large number of neurons that are tuned to different spatial frequencies. Particularly, in this paper we consider a set of neurons within the spatial-frequency hypercolumn, which is found in the primary visual cortex of primates [16]. In perceptual discrimination tasks, the observer is required to infer the statistical parameters from the neuronal population activities. The activation patterns elicited over the neural population take different shapes depending on the underlying statistical parameters (such as 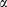) of input visual stimuli, and this gives clues for discriminating the stimulus parameters. Discrimination by the real nervous system, however, suffers from noise in neuronal firing. As is widely accepted, the firing rate of a neuron fluctuates from trial to trial, typically showing a Poisson-like variability [17], [18]. Because of the uncertainty in the neuronal encoding process, the inference from it is expected to contain errors at some rate even if we assume that the stimulus information is optimally read out by an ideal observer in the subsequent decoding stage. Human performance, which is a consequence of neuronal population activity, is expected to be related to the resolution of the neuronal information representation; however, it is not clear what part of the neuronal encoding process critically affects the human behavioral data. To elucidate this point, we quantify the modeled neural encoding accuracy in terms of Fisher information. Specifically, here we suggest that the distributional property of neurons should be taken into account for explaining real data.

The remaining part of this paper is laid out as follows. In the Model section, we formulate the encoding processes of the amplitude spectrum slope by the nervous system. The model predicts that the frequency-tuned neurons have asymmetric tuning curves for 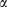. We also introduce Fisher information as a measure of decoding accuracy based on stochastic neuronal population activities and suggest how that measure relates to experimentally observed human performance. In the Results section, we compare the theoretical discrimination performance given by the present model to recently reported psychophysical data. The model explains the reported qualitative and quantitative differences in human performance between the fovea and the parafovea. We show that the neuronal distribution profile plays an important role in the emergence of the fovea-parafovea difference. Finally, we discuss the relation to other computational models, the extension of the current computational framework, the biological plausibility, and possible experimental tests for the current model.

## Methods

### Encoding the slope of the amplitude spectrum falloff and neuronal ‘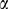-tuning’

**Figure 1:**
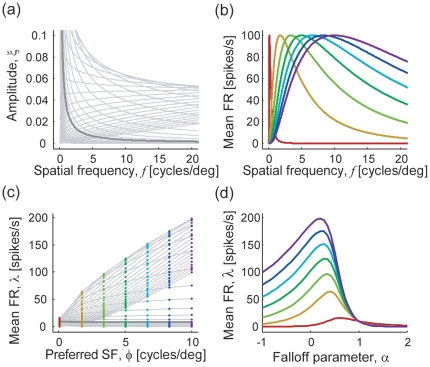
Modeling of visual stimuli and neuronal responses. (a) Models of amplitude spectra of images, where the amplitude spectrum of each image satisfies 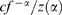. Each curve represents a spectrum with a particular falloff parameter 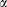 from −1 to 2. Contrast-determining parameter 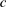 was set to 0.05 for each image. The thick curve represents the spectrum of an image with 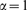. (b) Spatial frequency tuning of model neurons within a hypercolumn. Seven example neurons with most-preferred frequencies are spaced evenly from 0.01 to 10 cycles/deg (colored from red to indigo). The tuning curves are modeled with log Gaussian functions. (c) Demonstration of population activity evoked by the images satisfying the power law with different 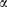 values. Dot colors are matched to those in panel b. Each curve links the unit activities evoked by a common stimulus. The thick flat line represents the responses to the image with 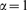. (d) Hypothetical response curves as functions of 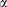, derived from activity profiles shown in panel c.

Natural images are broad-band stimuli. Their amplitude spectra represented in the Fourier domain are known to follow the power law.
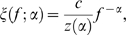
(1)where 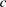 is the parameter determining the contrast of the image and 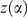 is the normalization constant,
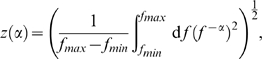
(2)which can be written in an explicit form as
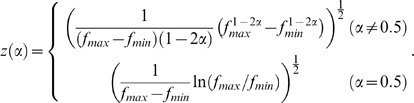
(3)The parameters used here and other important parameters used in this section is summarized in Table 1. The images represented as Eq. (1) have the same root mean square (RMS) values as the contrast measure. Figure 1a shows the models of input images with different spectra falloff parameters 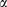. The thick curve in the figure represents the spectrum where 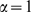, which is typical for natural scene imagery. In the present study, we only consider spatially isotropic visual images for the simplicity.

**Table 1:** Summary of the variables and the functions used in the text.

Symbols	Descriptions
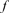	spatial frequency [cycles/deg]
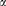	amplitude spectrum falloff parameter
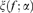	amplitude spectrum of the input image
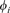	preferred spatial frequency of the 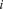 th cortical unit [cycles/deg]
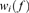	frequency tuning curve of the 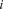 th cortical unit ( 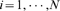 ) [spikes/s]
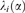	expected activity of the 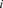 th cortical unit [spikes/s]
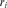	trial-to-trial firing rate of the 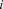 th cortical unit [spikes/s]
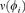	density of units with the preferred spatial frequency 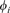 [cells  deg/cycles]

Neurons in the early visual cortex selectively respond to stimuli with different spatial frequencies. Much of the knowledge about neuronal tuning for spatial frequency has been gathered based on experiments employing single-wavelength stimuli (i.e., sine wave gratings). Those stimuli are represented with the delta function in the spatial frequency domain, and the frequency tuning functions of neurons are interpreted as the impulse responses. Following the conventional notion of log-scaled spatial-frequency selectivities and a bank of log Gabor filters [3], [19]–[21], we model the spatial frequency tuning profile of unit 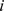 with a log Gaussian function, defined as
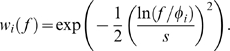
(4)In Eq. (4), we ignored the dependence of tuning bandwidth 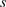 on the neuron's peak preferred frequency 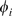 to simplify the model. Although physiological study suggests negative correlation between the bandwidth (in octave scale) and preferred frequency of neurons in the Macaque primary visual cortex [22], here we consider a limited range of neuronal preferred frequency (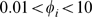, at most) in which the relation between those two parameters can be roughly approximated by a constant function. Figure 1b shows the frequency tuning functions of seven example model neurons within a spatial frequency hypercolumn.

We assume that the expected response of each neuron is roughly approximated by the dot product of its frequency tuning function and the image
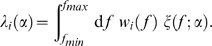
(5)Equation 5 represents the tuning over the spatial frequencies, regardless of the wave phases; it is considered as a model of complex cell responses, which are approximated by the sum of rectified activities of linearly responding cells (e.g., simple cells in the primary visual cortex, or alternatively the pooled summation of rectified outputs of simple cells preferring the unique spatial frequency and different spatial phases (although we should note that the variability in the pooled spike counts of a simple cell population is generally not equal to that of the sole response by a complex cell). We also note that the set of model unit outputs 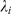 does not provide a complete representation of the input image itself; that is, we cannot discriminate two images with the same amplitude spectrum based only on 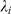
s. To discriminate them, another set of neurons that are sensitive to the phases of Fourier components (e.g., simple cells) are needed. In the present study, the purpose of which is to computationally investigate the relationship between 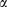 values and the neural responses, we do not focus on those additional set of neurons. Another note concerns the two-dimensional nature of neuronal receptive fields: strictly, Eq. (5) needs to contain integration not only over the spatial frequency but also over the orientation, considering the neuron's orientation tuning. In the present study, however, we omit the integration over the orientation since we consider spatially isotropic visual images. Regarding this issue, note that the precise form of the function inside the integral of Eq. (5) depends on how we model the neuron's two-dimensional tuning over the orientation and the spatial frequency. To the best of our knowledge, however, there is not physiological consensus about what a neuronal orientation tuning is like for 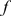 that deviates from the preferred spatial frequency, and we adopt the most simple formulation as in Eq. (5), which is comparable to the previously proposed models [3], [21], [23]–[25].

From Eqs. (1), (4) and (5), we have (see 1 for the derivation)
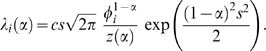
(6)
Figure 1c shows the population activity evoked by images satisfying the power law with different 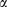 values. Of particular note, Eq. (6) suggests that the responses 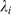 are constant across the whole units when 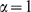, as illustrated by the thick flat line in Fig. 1c. Equation (6) represents the cell's tuning profile concerning the steepness of the amplitude spectra falloff (sometimes called ‘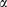-tuning’). This property has been pointed out previously by other authors [3], [21], [23]–[25]. Figure 1d shows the hypothetical response curves as functions of 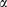. Whether neurons actually have tunings for 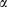 is testable by conducting electrophysiological recoding with appropriate set of visual stimuli.

Equation 5 assumes a linear relationship between the neural response and the amplitude in the visual stimulus. It might be an oversimplified model, considering nonlinearities in the actual cortical neurons. Nevertheless, it is useful to check what is reproduced (or failed to be reproduced) by the model for understanding its characteristics. We will see later that such a simple model can replicate, at least in a qualitative aspect, the experimentally observed complexities of human psychophysical ability to discriminate changes in amplitude spectrum slope. We also consider a model that takes into account the neural response nonlinearities as introduced in the next subsection.

### Neuronal interaction within a hypercolumn

Equation (5) ignores interaction among the units. In an actual cortex, however, there seems to be a gain control process within the hypercolumn that reduces the difference between the unit responses to high and low contrast natural images [26]. To account for the neuronal interaction, contrast normalization models [27]–[29] often suppose divisive modulation of cell responses with the gain determined by the pooled activity of a large neural population in the cortical neighborhood (within the hypercolumn). Here we divide each unit response 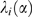 (in Eq. (6)) by a power of the pooled neuronal activity within the hypercolumn so that the model represents the response gain control in the neuronal population:
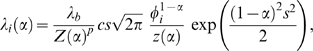
(7)where 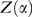 is a divisive normalization term that is defined by
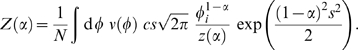
(8)Here we introduced a density function 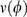, which describes the distribution of units over the preferred spatial frequency 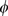. The constants 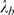 and 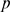 in Eq. (7) are the model parameters that control the order of mean neural activity and the strength of the gain control, respectively. Note that Eq. (7) is reduced to Eq. (6) by setting 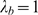 and 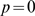.

### Decoding

We assume the Poisson spiking of each unit, where the log likelihood of the falloff parameter 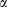 can be written in terms of the hypothetical mean firing rates 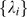 as
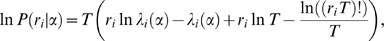
(9)where 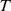 is the time interval during which the spikes are sampled (see 2 for the derivation of Eq. 9). If we assume the independent spiking of the units, the likelihood based on the responses of the whole unit is given as
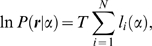
(10)where 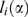 represents the term in parentheses on the right-hand side of Eq. (9). We consider the maximum likelihood estimator for the falloff parameter, given as
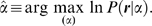
(11)


As a measure of the variability in the estimate 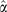, we can use the Fisher information of the neural population code [30]–[34]. Fisher information enables us to quantify how accurately the subject can distinguish two stimuli having slightly different values of amplitude spectrum slopes 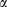 and 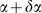. Moreover, Fisher information is mathematically related to experimentally observable values such as subject's discrimination threshold [31], [33] (see, for example, [34] for an introduction to the use of Fisher information in a framework of neural population coding). For the assumption of unbiased estimation, the inverse of the Fisher information gives a lower bound for the variability on 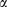 through the Cramér-Rao inequality:
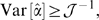
(12)where 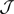 denotes the Fisher information concerning 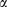. For the assumption of independence of each neuronal firing, the Fisher information can be written in a factorized form as
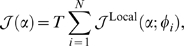
(13)where
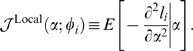
(14)The value 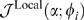 in the above equation represents the *local* Fisher information per unit and per second. Performing the necessary calculations (see Appendix S3) yields
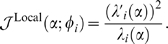
(15)The value on the left-hand side of the above equation can be interpreted as (the square of) the signal to noise ratio. At a sufficiently large population size 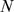, the summation in Eq. (13) can be substituted by the integral over the neural distribution:
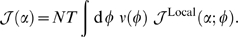
(16)


### Discrimination threshold

Here we summarize the relationships between the Fisher information and the experimentally obtained measures of human performance, assuming a particular psychophysical setting. We consider discrimination with two-alternative forced choice, in which each trial presents two stimuli in random order; the two stimuli have falloff parameters that are slightly different from each other (say, 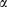 and 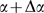). The subject's task is to determine which of the two had the larger (or smaller) parameter (a series of psychophysical studies proved that human subjects can perform this kind of task, [10]–[12], [35]). For a large population of neurons, the subject correct rate (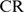) for the discrimination task around a particular falloff parameter 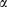 is given by
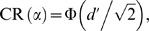
(17)where 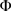 denotes the cumulative distribution function of the standard normal distribution:
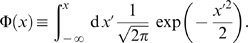
(18)The psychophysical measure of the discriminability 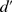 between two values 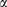 and 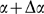 is represented in terms of Fisher information as
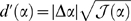
(19)when the number of neurons is sufficiently large (e.g., [31], [33]). From Eqs. (19) and (19), we compute the discrimination threshold at 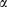 with a given threshold value setting for the correct rate, 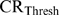, as
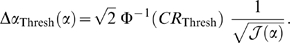
(20)Note that the discrimination threshold is inversely proportional to the square root of the Fisher information 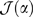, regardless of the setting of 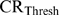.

## Results

**Figure 2:**
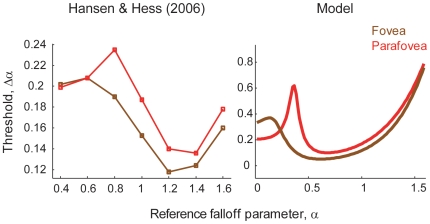
Empirically and theoretically obtained thresholds for discriminating falloff parameters 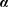. (Left panel) Data extracted from Hansen and Hess [8]. (Right panel) The discrimination thresholds predicted by the current models of multiresolutional population codes. Brown and red curves show the thresholds for fovea and parafovea, respectively.

In this section, we check the plausibility of our model by comparing its performance to experimentally obtained data. Human performance at discriminating the amplitude spectrum falloff parameter 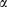 has been a topic of debate in visual psychophysics [8], [10]–[12] since the first investigation conducted by Knill *et al.* [10]. Hansen and Hess [8] established that human performance at discriminating 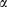 differs quantitatively and qualitatively depending on the retinal position (i.e., the fovea or the parafovea) at which stimulus patches are presented. The difference between the fovea and the parafovea is most apparent for stimuli with relatively small 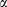 values (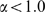). For the parafovea, the discrimination threshold peaks at a small 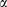 whereas no peak is observed for foveal presentation, as the data show in Fig. 2a. It has not been explained by previously proposed computational frameworks why there is such a qualitative disparity between the fovea and the parafovea, and why the sensitivity in the parafovea forms an N-shaped curve [8]. We show that this aspect of the fovea-parafovea difference can be explained with the current model, assuming different cell distributions 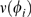 in Eq. (16) for the fovea and for the parafovea.

### Distribution of neurons explains fovea-parafovea difference

**Figure 3:**
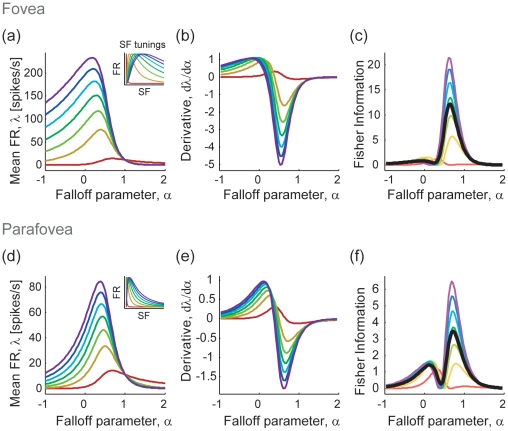
Derivations of Fisher information carried by neural populations. (a–c) Fovea. Preferred spatial frequencies of the units varied from 0.01 to 10 cycles/deg. (a) Hypothetical 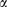-tuning curves (identical to Fig. 1d). Inset illustrates the spatial frequency tuning curves and the distribution of the preferred frequencies of the model neurons (same as shown in Fig. 1b). The figure shows seven example units with preferred frequencies spaced evenly from 0.01 to 10 cycles/deg (colored from red to indigo). (b) The derivative functions of the 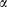-tuning differentiated by the falloff parameter (i.e., 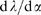). (c) The *local* Fisher information of the individual units (thin colored curves) and their average (i.e., information per unit; thick black curve). Colors of curves in panels b and c are matched to those in panel a. (d–f) Same as (a–c), but computed for the parafovea, where the units' preferred spatial frequencies varied from 0.01 to 1 cycles/deg. Seven example units, whose preferred frequencies are spaced evenly from 0.01 to 1 cycle/deg (colored from red to indigo).

For simplicity, we first ignore the neuronal interaction within the hypercolumn (i.e., 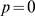 in Eq. (7)). Here, we show that this simple model is enough to reproduce the complexity of human performance at 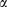 discrimination. The effect of neuronal interaction is considered in the next subsection by comparing the model performance for two cases, 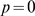 and 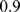. To get an intuitive understanding of how the qualitative difference between the fovea and the parafovea arises in the model, it is illustrative to follow the process by which the Fisher information is derived from the 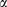-tuning profile of the neural population. Figure 3 depicts the procedure for computing the Fisher information from the neuronal tuning curves. The panels a–c and d–f in the figure show the cases for the fovea and the parafovea, respectively. The model parameters were set to be the same for both conditions except that the fovea had a broader distribution of the unit's preferred spatial frequency, covering a relatively higher frequency domain than the parafovea; this assumption is in qualitative agreement with an electrophysiological study that intensively investigated the spatial frequency selectivity of Macaque striate cells with various loci of receptive fields [22]. The peak preferred spatial frequencies of the model units varied from 0.01 to 10 cycles/deg for the fovea and from 0.01 to 1 cycles/deg for the parafovea, with the total number of units 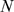 kept the same between those two conditions. Here we simply assumed uniform distributions for both cases; more detailed estimations of the neuronal distributions are considered in the next subsection. Figures 3a and d, respectively, show the 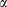-tuning curves, 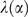, of seven example model neurons in the fovea and the parafovea (the neurons in panel a are identical to those shown in Fig. 1d). Note that their vertical scales are different. The inset figures show the spatial frequency tuning curves of each of seven neurons, illustrating the difference in the distributions of the peak preferred frequencies between the fovea and the parafovea. Figures 3b and e show the derivatives of the tuning functions differentiated by 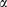 (i.e., 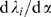). The absolute values of those derivative functions represent the sensitivities of the units to a slight fluctuation around particular 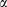 values. The Fisher information measures are given by computing the ratio of the derivative and the mean firing rate, as we have seen in Eq. (15). Figures 3c and f show the Fisher information carried by each individual unit and the whole population. Each of the thin colored curves in the figures represents the contribution of each unit 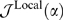 to the total Fisher information 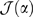. The thick black curves represent the information averaged over the population (i.e., 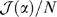). Notably, in the parafoveal condition the average Fisher information shows clear double peaks (the primary peak around 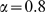 and the secondary peak around 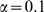), whereas the secondary peak is almost ignorable in the foveal condition.

Figure 2 compares the empirically and theoretically obtained thresholds for discriminating the falloff parameter 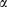. The left panel of Fig. 2 shows the data cited in Hansen and Hess [8]. The brown and the red curves in the figure represent the discrimination thresholds for the fovea and the parafovea, respectively. The right panel of Fig. 2 shows the discrimination thresholds computed with the current models. Clearly, the model captures the qualitative characteristics of the real data. Most importantly, the threshold peak in the parafoveal condition at a small 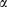 is replicated with the model. Because the discrimination threshold 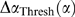 is given by a decreasing function of the Fisher information as Eq. (20), the existence of double peaks of the information in the parafovea (Fig. 3f) indicates the presence of two threshold minima with the maximum between them. On the other hand, in the foveal condition, only a faint threshold peak is observed, reflecting that the secondary peak of Fisher information is much more moderate than the primary peak (Fig. 3c). Although the model replicates the qualitative structure of the experimental data, there are some quantitative differences. The model is inefficient for the foveal threshold at 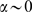, and for the foveal and parafoveal threshold at 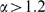. Also, the early peak in parafoveal threshold appears at an alpha that is smaller than the data, and the peak height from the flat region preceding it is much higher in the model than in the data. In the next subsection, we show that these quantitative departures from the data is diminished when we use the model taking into account the response gain control within the hypercolumn.

### Model fitting and estimation of cell distribution

The observation in the previous subsection shows that the simplified model, which ignores the effect of neuronal interaction, succeeds in qualitative reproduction of the experimental data. This indicates that the neuronal interaction need not be primarily considered for explaining the human sensitivity to the changes in 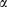 value, especially when we focus on the origin of its qualitative structures such as the fovea-parafovea difference and N-shaped sensitivity curve seen in the parafovea. On the other hand, the interactions among neurons should be taken into account when we aim at simulating more realistic reactions in the cortex. In this subsection, we consider how neuronal interaction within a hypercolumn affects the performance of the model. The neuronal responses to stimuli presented at suprathreshold contrasts are attenuated by inhibitory connections in the *contrast gain control* process [36]–[38]. It is widely accepted that the perceptual ability to discriminate grating contrast is enhanced or degraded by a surrounding or superimposed spatial context, depending on the stimulus contrast, orientation and spatial frequency [39]–[56].

**Figure 4:**
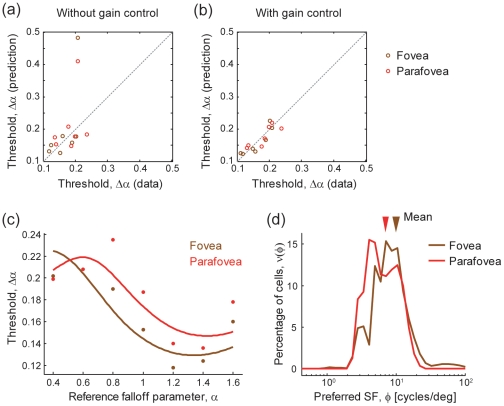
Estimating neuronal distributions so that they fit the model prediction with the data. Data are the same as in Fig. 2. (a,b) Model predictability (a) without and (b) with gain control within hypercolumn. In both models, we fitted the data by varying the numbers of neurons as the fitting parameters. (c) Data fitting by model that takes into account gain control within hypercolumn. (d) Estimated neuronal distributions using the model with gain control. The arrows above the histograms indicate the mean preferred spatial frequency of neurons within foveal (brown) or parafoveal (red) hypercolumns. We set the model parameters 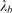 and 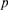 in Eq. (7) as 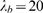 and 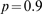.

**Figure 5:**
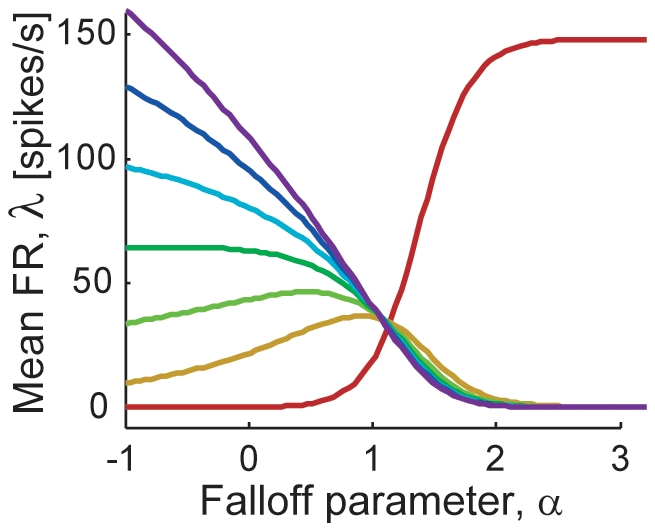
Hypothetical response curves as functions of 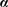 with model that takes into account gain control within hypercolumn. The seven representative neurons in the foveal condition, in which the neuronal distribution is estimated as shown in Fig. 4d (brown histogram). The most-preferred frequencies of sampled neurons are spaced evenly from 0.01 to 10 cycles/deg (colored from red to indigo).

We tried two parametrically different models for fitting the experimental data: one with response gain control within a hypercolumn (implemented by setting 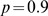) and the other without (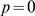). Here, we again used the experimental data of Hansen and Hess shown in Fig. 2 and varied the neuronal distribution 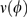 as the model parameters to replicate the human subject performance. To make the problem numerically solvable, we discretized the neuronal distribution; in both models, we divided the preferred spatial frequencies into 50 bins that are evenly arrayed in log scale and varied the number of neurons that prefer spatial frequencies within each bin as a parameter. Note that both of the models have the same number (i.e., 50) of free parameters. Figures 4a and b compare the fitting performance of the two models. We found that, for the present problem settings, the model with response gain control provides better fitting performance than the one without. This is because the model with gain control more precisely locates the peaks and dips of the plot, although both models can reproduce the qualitative structures (fovea-parafovea difference and N-shaped threshold curve in the parafovea) of the subject's sensitivity. In Fig. 5c, the experimental data and the fitted curves using the model with gain control are shown. Figure 5d depicts the neuronal distributions in fovea and parafovea estimated by fitting the data to the model with gain control. The estimated neuronal distributions in the fovea and parafovea are different. Consistent with the observation in the previous subsection and an electrophysiological study [22], the foveal neurons are estimated to have a tendency to prefer higher spatial frequency than the parafoveal neurons.

**Figure 6:**
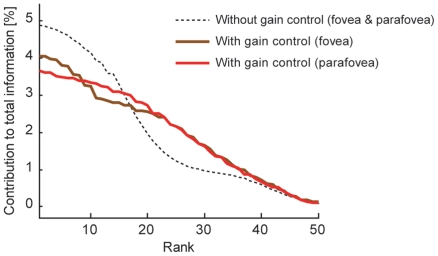
The unit contribution to the total Fisher information carried by the whole population, ranked according to the proportions of contribution. For each unit, we calculated the average of unit-wise Fisher information 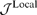 within 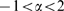, and then analyzed the averaged contributions for 50 neurons having different preferred spatial frequencies between 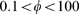, which are the same as those used in the model fitting in Fig. 5. When compared to the case for no neuronal interaction (gray line), the models with response gain control within the hypercolumn suggest more broad distributions of information both in fovea (brown) and parafovea (red). Note the slightly different result between fovea and parafovea when considering gain control among neurons, because the distribution of cell number 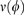 affects the 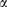-tuning curves of the individual units.

Taking into account the neuronal interaction within the hypercolumn causes a subtle change in the neuronal 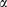-tuning and information distribution over the cell population. In the model without neuronal interaction, as shown in Figs. 1c and d, the dynamic ranges of neurons largely differed between those with high (red) and low (indigo) preferred spatial frequencies. This originates from the difference in their tuning widths; that is, high-frequency-selective neurons have broader tuning widths than low-frequency-selective ones as in Fig. 1b), and are more dramatically affected by changes in the shape of the amplitude spectrum. From Fig. 1d, it seems that most of the neuronal firing finish by the time alpha gets to one; although there *are* neurons having response peaks around 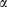 = 1 (such as the neuron shown by the red tuning curve in Fig. 1d), their responses are not conspicuous because of the large difference in their response dynamic ranges. In addition, the difference in the dynamic ranges resulted in non-uniformity in the quantities of information carried by individual neurons in the current model, as shown in Figs. 3c and f; the figures indicate that the high-frequency-selective neurons, generally, carry much more information than low-frequency-selective ones. Figure 5 shows the 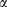-tuning curves of units with the same preferred spatial frequencies as in Fig. 1d but derived with response gain control within the hypercolumn. The gain control among the units reduces the difference in the neuronal dynamic ranges. We can see the units having larger responses around 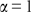, compared to the case in which the gain control was not taken into account. Also, it works to uniformize the unit contributions to the total information transmission. Figure 6 demonstrates how the information distribution is changed by incorporating the response gain control among neurons. The abscissa shows the ranks of units ordered according to amount of contribution to the total value of Fisher information; the ordinate indicates the unit-wise share of the contribution (percentage of total information conveyed by the whole population), which is given here as the average of local Fisher information 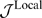 within 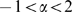 (the change in the averaging range does not affect the quality of result). We sampled the 50 model neurons with different preferred spatial frequencies that are considered in the above, and used the distributions of cell numbers estimated from the experimental data (Fig. 4d). Without gain control, the great extent of information is carried by the first 20 units (dashed curve). In contrast, model neurons with gain control show longer-tailed distributions (brown and red curves), indicating that the stimulus information is shared by neurons with a wide variety of preferred spatial frequencies. An evident advantage of such a distributed representation of information is its robustness to cell death. The neurons preferring similar spatial frequency are located near each other as they make the columnar structures in the cortex [16], and they can be simultaneously damaged by injury or obstruction of blood vessels. Information sharing by neurons in various spatial frequency columns is expected to reduce the risks of information loss caused by such biological damage.

## Discussion

We have proposed a computational model of neuronal encoding and decoding of natural image statistics. We hypothesized the elicited population activities of spatial-frequency selective neurons, which are observed in the early visual cortex. This work provides the first clear modeling of neurophysiological substrates concerning human perceptual resolution for discriminating the amplitude spectrum slopes in natural scenes. We have also shown that human sensitivity for the discrimination of the spectrum falloff parameter 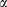 suggested by the current model is consistent with experimental data. The model suggested that the differences between performance in fovea and parafovea could be explained by taking into account the distributional difference of cortical neurons over preferred frequencies. Further, the model predicted that the neurons selective to spatial frequencies have asymmetric and fat-tailed tuning curves for the amplitude spectra slopes. This prediction on the ‘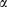-tuning’ of the frequency-selective neuron can be directly tested by physiological experiments.

In the current model, the distributional properties of neuronal stimulus preferences play a critical role in reproducing the qualitative features seen in human psychophysical performance. Furthermore, fitting result of the experimental data shows that the model taking into account the intra-hypercolumn gain control explains well the human performances from the quantitative view points. The cell distributions estimated from the data suggested that the neurons in the fovea tends to prefer higher spatial frequency than in the parafovea, which is consistent to the physiological insight in animal cortex. The effects of a change in neuronal distribution have not been a central topic in conventional modeling studies. However, the present results clearly demonstrate that precise estimation of the neuronal distribution is important for linking microscopic neurophysiological phenomena and macroscopic human behaviors.

### Relation to other computational models

In this paper, we modeled neuronal representations of image statistical values (amplitude spectrum slopes) assuming a population of cells with various preferred spatial frequencies. The similar idea of multi-resolution representation of natural image was previously proposed by Párraga et al. [57] in a different context. They reported a success of the multi-resolution model in explaining human psychophysical ability to discriminate small changes in morph sequences of natural objects with various amplitude spectrum slopes. Their and our models differ in several points: first, it was not revealed in the previous work whether and how the value of amplitude spectrum slopes themselves are distinguished based on the multi-resolution representation. Second, they did not provide explicit model of the neuronal firing stochasticity, and thus it was difficult to interpret the human perceptual performance in terms of the variability in neuronal coding. In the present study, assuming the Poisson spike generation, we have related the stochastic neural activity to the human perceptual ability through the Fisher information as a measure of the neural encoding accuracy. Third, in previous study it was also not clarified how the cell distribution characterizes of the encoding performance by a whole cell population in the amplitude spectrum discrimination. We have found that the expected fovea-parafovea difference in the cell distribution over the preferred spatial frequency would cause a qualitative disparity among the psychophysical skills in the fovea and the parafovea, which was not demonstrated previously.

Another topic concerns the population coding that represents shapes of functions. Because a natural image has a broad spectrum over the spatial frequency, the neuronal response to it can be seen as representing not a single value but a function, such as 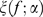. The population coding that represents a function is known as *distributional population code* [58], [59]. While the original distributional population code aims to reconstruct the entire form of the function, our current focus is to infer its control parameter 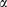. In the present study, we simply assumed that the amplitude spectrum of natural images exactly followed the power law. This is equivalent to assuming an infinitely thin prior probability distribution over the spectrum function 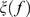, that is,
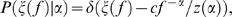
(21)where 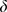 is the delta function. Under this assumption, the neuronal responses to a natural image are represented by a standard population code that encodes 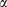 but not 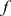. An actual natural stimulus, of course, has some fluctuation in the value of 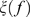 around its mean 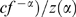, so we can consider its distribution 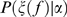 with nonzero variability. In this case, the inference of the falloff parameter 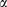 corresponds to *hyperparameter estimation* in the framework of a hierarchical Bayesian estimation.

### Extension of the current work

One possible direction for extension of the current model would be introducing a prior distribution over 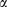. Under such a Bayesian decoding scheme, the estimated 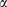 values are biased by the prior distribution, which represents the knowledge about image statistics observed in a natural environment (e.g., 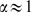). An interesting issue is to consider the subject's perception of the image in those situations. Visual image reconstruction based on knowledge about typical natural image statistics can provide powerful explanations for a wide range of brightness illusions [21].

Another direction is modeling more complex structures of natural scene statistics. This would be important especially when we consider image categorization. For example, Torralba and Oliva [60], [61] proposed more elaborate computational algorithms for image categorization using image statistics of natural scenes. In those models, the stimulus statistics are characterized by a greater level of detail, including asymmetries among the shapes of spectra falloff in different directions (i.e., vertical or horizontal, etc.). At the present stage, little is known about whether actual nervous system computes such an advanced type of image statistics. Taking into account the stochasticity of neuronal firing, more complex model of image processing, rather than the calculation based on the simple power law, would be related to human performance within the same framework.

### Experimental predictions

Our model provides two predictions that are both testable. First, the model predicts changes in the discrimination threshold following neuronal sensitivity modulation caused by adaptation or spatial context. For example, adapting to high frequency stimulus is considered to decrease neuronal response gains of high-frequency selective neurons. The decrease in gain caused by adaptation mimics a decrease in the number of neurons that prefer high frequency stimuli. Second, the model also predicts that we will have different experimental results when we use visual stimuli that do not follow the power law (e.g., images blurred by Gaussian filters). Subject sensitivities for discriminating such *unnatural* stimuli can show qualitative differences from those for natural stimuli. For example, spatial blurring (or low-pass filter) removes the high-frequency signals from images; under the assumption of linearity, this manipulation emulates a decrease in the number of high-frequency selective neurons in the cortex, like what is expected to occur in visual adaptation (note that this blurring procedure is not equal to just varying the 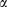 value because the amplitude spectrum shape deviates from the exponential function after this manipulation). Therefore, especially in the fovea, we might see the emergence of a parafovea-like peak in the discrimination threshold when we use blurred natural images.

## Supporting Information

Appendix S1 Derivation of Eq. 5.
(0.02 MB PDF)

Appendix S2 Derivation of Eq. 9.
(0.02 MB PDF)

Appendix S3 Derivation of Eq. 15.
(0.02 MB PDF)
