# Supplementary material for: Discriminating Natural Image Statistics from Neuronal Population Codes
Source: PLoS One. 2010 Mar 25;5(3):e9704. doi: 10.1371/journal.pone.0009704 (PMC2845616; doi:10.1371/journal.pone.0009704)
Supplement: Appendix S1 — Derivation of Eq. 5. (0.02 MB PDF) [file pone.0009704.s001.pdf]

Appendix S1. Derivation of Eq. 5

We consider the case in which $f_{min} = 0$ and $f^{max} \to \infty$. Substituting Eqs. 1 and 4 into Eq. 5,

$$\lambda_i(\alpha) = \int_0^\infty \mathrm{d}f \ \exp\left(-\frac{1}{2}\left(\frac{\ln(f/\phi_i)}{s}\right)^2\right) \frac{c}{z(\alpha)} f^{-\alpha}.$$

Denoting $\ln(f/\phi_i)/s$ as y, since $f = \phi_i e^{sy}$ and $\mathrm{d}f = s\phi_i e^{sy}\mathrm{d}y$,

$$\begin{aligned}\lambda_i(\alpha) &= cs\frac{\phi_i^{1-\alpha}}{z(\alpha)} \int_{-\infty}^{\infty} \mathrm{d}y \ \exp\left(-\frac{y^2}{2} + (1-\alpha)sy\right) \\ &= cs\frac{\phi_i^{1-\alpha}}{z(\alpha)} \int_{-\infty}^{\infty} \mathrm{d}y \ \exp\left(-\frac{1}{2}\left(y-(1-\alpha)s\right)^2 + \frac{(1-\alpha)^2 s^2}{2}\right).\end{aligned}$$

Finally, we have

$$\lambda_i(\alpha) = cs\sqrt{2\pi}\,\frac{\phi_i^{1-\alpha}}{z(\alpha)} \exp\left(\frac{(1-\alpha)^2 s^2}{2}\right).$$

Note that, in reality, for $f^{max} \to \infty$ the normalization constant $z(\alpha)$ takes a near-zero or an extremely large value depending on the value of α; in this paper, we consider the above calculation as an approximation of the situation in which the image resolution (i.e., the maximum spatial frequency) is sufficiently large.
