# Supplementary material for: Discriminating Natural Image Statistics from Neuronal Population Codes
Source: PLoS One. 2010 Mar 25;5(3):e9704. doi: 10.1371/journal.pone.0009704 (PMC2845616; doi:10.1371/journal.pone.0009704)
Supplement: Appendix S2 — Derivation of Eq. 9. (0.02 MB PDF) [file pone.0009704.s002.pdf]

Appendix S2. Derivation of Eq. 9

We have a set of firing rates $\boldsymbol{r}$ for the N neurons in each trial ($\boldsymbol{r} = [r_1, \cdots, r_N]$). In the present model we assume that each neuron fires independently, and then the joint probability for the whole population is given by

$$P(\boldsymbol{r} \mid \alpha) = \prod_{i=1}^{N} P(r_i \mid \alpha).$$

The number of spikes for each neuron varies across trials. We assume that this variability is described by a Poisson process:

$$P(r_i \mid \alpha) = \frac{(\lambda_i T)^{r_i T}}{(r_i T)!} e^{-\lambda_i T},$$

where T is the length of time during which spikes are sampled (i.e., $r_i T$ spikes are sampled in total during the observation). Therefore, the log likelihood of the falloff parameter α is written as

$$\ln P(r_i \mid \alpha) \quad = \quad T \left(r_i \ln \lambda_i(\alpha) - \lambda_i(\alpha) + r_i \ln T - \frac{\ln\left((r_i T)!\right)}{T} \right).$$
