# Supplementary material for: Discriminating Natural Image Statistics from Neuronal Population Codes
Source: PLoS One. 2010 Mar 25;5(3):e9704. doi: 10.1371/journal.pone.0009704 (PMC2845616; doi:10.1371/journal.pone.0009704)
Supplement: Appendix S3 — Derivation of Eq. 15. (0.02 MB PDF) [file pone.0009704.s003.pdf]

Appendix S3. Derivation of Eq. 15

In Eq. 10, we defined l_i as

$$l_i = r_i \ln \lambda_i(\alpha) - \lambda_i(\alpha) + r_i \ln T - \frac{\ln((r_i T)!)}{T}.$$

The first and the second derivatives of l_i are respectively given as follows:

$$\begin{aligned} \frac{\partial l_i}{\partial \alpha} &= r_i \frac{\lambda_i'(\alpha)}{\lambda_i(\alpha)} - \lambda_i'(\alpha), \\ \frac{\partial^2 l_i}{\partial \alpha^2} &= r_i \frac{\lambda_i''(\alpha)}{\lambda_i(\alpha)} - r_i \frac{(\lambda_i'(\alpha))^2}{(\lambda_i(\alpha))^2} - \lambda_i''(\alpha). \end{aligned}$$

Substituting it into Eq. 14 yields the *local* Fisher information:

$$\begin{aligned} \mathcal{J}^{\mathrm{Local}}(\alpha; \phi_i) &\equiv E\left[-r_i \frac{\lambda_i''(\alpha)}{\lambda_i(\alpha)} + r_i \frac{(\lambda_i'(\alpha))^2}{(\lambda_i(\alpha))^2} + \lambda_i''(\alpha) \middle| \alpha \right]. \\ &= -\lambda_i(\alpha) \frac{\lambda_i''(\alpha)}{\lambda_i(\alpha)} + \lambda_i(\alpha) \frac{(\lambda_i'(\alpha))^2}{(\lambda_i(\alpha))^2} + \lambda_i''(\alpha). \\ &= \frac{(\lambda_i'(\alpha))^2}{\lambda_i(\alpha)}. \end{aligned}$$
